# Impact of Phage Therapy on Multidrug-Resistant *Escherichia coli* Intestinal Carriage in a Murine Model

**DOI:** 10.3390/microorganisms9122580

**Published:** 2021-12-13

**Authors:** François Javaudin, Pascale Bémer, Eric Batard, Emmanuel Montassier

**Affiliations:** 1 MiHAR Laboratary, EE1701, University of Nantes, 44200 Nantes, France; pascale.bemer@chu-nantes.fr (P.B.); eric.batard@univ-nantes.fr (E.B.); emmanuel.montassier@chu-nantes.fr (E.M.); 2 Emergency Department, Nantes University Hospital, 44000 Nantes, France; 3 Department of Bacteriology, Nantes University Hospital, 44000 Nantes, France

**Keywords:** phage therapy, extended-spectrum beta-lactamase, drug resistance, multidrug-resistant bacteria, intestinal carriage, enterobacteriaceae, *Escherichia coli*

## Abstract

Introduction: The growing resistance of bacteria to antibiotics is a major global public health concern. An important reservoir of this resistance is the gut microbiota. However, limited data are available on the ability of phage therapy to reduce the digestive carriage of multidrug-resistant bacteria. Materials and methods: Four novel lytic phages were isolated in vitro for efficacy against an extended-spectrum beta-lactamase-producing (ESBL) *Escherichia coli* strain also resistant to carbapenems through a carbapenemase OXA-48. The first step was to develop models of ESBL *E. coli* digestive carriage in mice. The second step was to test the efficacy of an oral and rectal phage therapy (a cocktail of four phages or microencapsulated phage) to reduce this carriage. Results: The two most intense models of digestive carriage were obtained by administering amoxicillin (0.5 g·L^−1^) continuously in the drinking water (Model 1) or pantoprazole (0.1 g·L^−1^) continuously in the drinking water, combined with amoxicillin (0.5 g·L^−1^), for the first 8 days (Model 2). Oral administration of the phage cocktail to Model 1 resulted in a transient reduction in the concentration of ESBL *E. coli* in the faeces 9 days after the bacterial challenge (median = 5.33 × 10^8^ versus 2.76 × 10^9^ CFU·g^−1^, *p* = 0.02). In contrast, in Model 2, oral or oral + rectal administration of this cocktail did not alter the bacterial titre compared to the control (area under the curve, AUC, 3.49 × 10^9^; 3.41 × 10^9^ and 3.82 × 10^9^ for the control, oral and oral + rectal groups, respectively; *p*-value > 0.8 for each two-by-two group comparison), as well as the administration of an oral microencapsulated phage in Model 1 (AUC = 8.93 × 10^9^ versus 9.04 × 10^9^, *p* = 0.81). Conclusions: Oral treatment with amoxicillin promoted digestive carriage in mice, which was also the case for the addition of pantoprazole. However, our study confirms the difficulty of achieving efficacy with phage therapy to reduce multidrug-resistant bacterial digestive carriage in vivo.

## 1. Introduction

The rise of bacterial resistance to antibiotics is considered a major public health concern worldwide. The World Health Organisation (WHO) has identified research on third-generation cephalosporins (3GC) and carbapenem-resistant *Enterobacteriaceae* as a key priority [1]. The gut microbiome is a major reservoir of antimicrobial resistance [2,3]. However, the healthy gut microbiota, under homeostatic conditions, can prevent colonisation by pathogens [4], but in the case of dysbiosis, induced by antibiotics, for example, intestinal colonisation by antibiotic-resistant agents is favoured [2]. Although this digestive carriage is asymptomatic, subjects colonised with extended-spectrum beta-lactamase-producing *Enterobacteriaceae* (ESBL-E) have a higher risk of developing an ESBL-E infection with increased morbidity, mortality, length of hospital stay and health care costs [5,6,7]. Furthermore, subjects with multidrug-resistant *Enterobacteriaceae* (MDR-E) intestinal carriage can contaminate their environment and thus can pass the resistant bacteria to others, especially in the hospital setting [8].

Different digestive decontamination strategies have been evaluated, but none have proved useful in routine practice [9]. However, new strategies could be interesting, such as phage therapy, but evidence is still lacking [10]. Oral phage therapy is considered safe in humans and has many advantages (target specificity, bactericidal activity, low environmental impact) [11,12]. Moreover, the mechanisms of bacterial resistance to antibiotics and phages are completely different, which means that a high level of antibiotic resistance in a bacterium is not necessarily associated with phage resistance [13,14]. Some animal studies have investigated the effect of phage therapy on intestinal carriage of *Enterobacteriaceae*, but one have been conducted on MDR-E [15].

Therefore, the aim of our study was to evaluate the ability of phage therapy to reduce the digestive carriage of MDR-E in a murine model.

## 2. Materials and Methods

### 2.1. Animals and Ethics Statement

Mice (6-week-old male C57BL/6J mice) provided by the Janvier laboratory (Le Genest-Saint-Isle, France) were used. The mice were housed at the Animal Research Centre of the Institute of Health Research 2, University of Nantes, and fed a controlled sterile diet in a controlled environment (12-h day/night cycle). Drinking water and food were provided ad libitum. The animals were isolated in individual cages with environmental enrichment to avoid cross-contamination by coprophagy. Their well-being was monitored daily.

The study was approved by the French Ministry of Higher Education and Research (APAFIS 11056) and by the Animal Ethics Committee of the Health Research Institute (reference 201708291549991).

### 2.2. Bacterial Strains and Gastric Gavage

A clinical strain of ESBL-producing *Escherichia coli* (*E. coli*), also resistant to carbapenems through a carbapenemase OXA-48, isolated from a surgical wound, was used to induce intestinal colonisation. Bacterial challenge was performed using 20-GA plastic feeding tubes (FTP-20-38, Instech Laboratories, Plymouth Meeting, PA, USA) and injecting 200 µL of solution into the gastric contents, containing a total of 10^6^ CFU of ESBL *E. coli*.

### 2.3. Phages and Gastric-Rectal Administration

The phages were isolated and selected by the Clean Cells company (Montaigu, France).

Microencapsulation of the PEC02 phage was performed by Kerry Richards and Danish J. Malik of the Department of Chemical Engineering of Loughborough University, Loughborough, UK, using a scalable membrane emulsification process [16].

Gastric administration was performed using 20 GA (Instech Laboratories, USA) or 18-GA (Instech Laboratories, Plymouth Meeting, PA, USA) plastic feedings tubes for encapsulated phages, followed by the injection of 200 µL of a solution containing a cocktail of the four phages (2.10^8^ PFU·mL^−1^) or PEC02 microencapsulated phages (1.10^6^ PFU·mL^−1^) into the gastric contents.

Rectal administration was performed using a 24-GA intravenous catheter (Terumo, Tokyo, Japan) and injecting 100 µL of solution containing a cocktail of the four phages (2.10^8^ PFU·mL^−1^) into the rectal contents. After rectal administration, mice were held in an inverted position for 30 s.

### 2.4. Murine Model of Intestinal Colonisation

**Figure 1:**
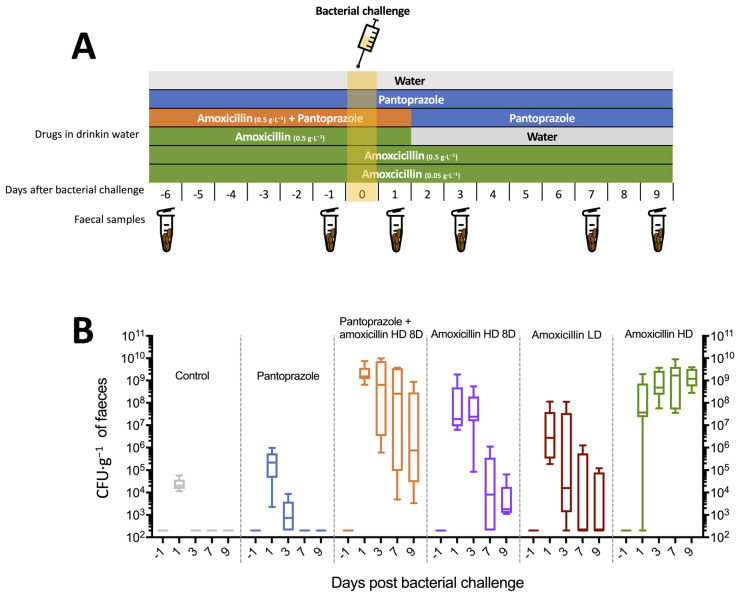
Murine model of ESBL *E. coli* intestinal colonisation (6 mice per group) (**A**), Experimental protocol. (**B**), Faecal concentration of ESBL *E. coli.*

Mice were exposed to different treatment regimens to induce intestinal dysbiosis. We used amoxicillin at concentrations of 0.5 g·L^−1^ (high dose, HD) or 0.05 g·L^−1^ (low dose, LD) and pantoprazole at 0.1 g·L^−1^, according to the schemes summarised in Figure 1, all in drinking water. We used injectable forms of these drugs in order to ensure that they were soluble in the water. Briefly, we tested six conditions: water, pantoprazole, pantoprazole + amoxicillin HD for the first 8 days, amoxicillin HD for the first 8 days, amoxicillin HD and amoxicillin LD throughout the experiment. The bottles were refilled every 3 days. There were six mice per condition tested. Bacterial challenge was performed after 7 days from the start of amoxicillin and/or pantoprazole. Stool sampling was performed at days 1, 6, 8, 10, 14 and 16.

### 2.5. Faecal Collection and Culture

On the day of sampling, each mouse was placed in a clean cage for 1 h to collect its faeces, which were immediately frozen at −80 °C. Each stool was weighed and then crushed (Mixer Mill MM 400, Retsch, Haan, Germany) with 1 mL of sterile saline water. Serial dilutions were performed, followed by manual inoculations of 100 µL onto ESBL selective chromogenic agar plates (ChromID ESBL, BioMérieux, Marcy-l’Étoile, France) before incubation at 37 °C for 24 h. ESBL *E. coli* were identified by the pink to burgundy coloration they take on these specific agar plates (Figure S1 in the Data Supplement).

### 2.6. In-Vivo Evaluation of Phage Therapy

#### 2.6.1. Experiment 1

Mice were exposed to amoxicillin treatment in the drinking water (0.5 g·L^−1^) throughout the experiment. Bacterial challenge was performed on day 7. From day 9 to day 11, six mice were treated daily by gastric gavage with the phage cocktail or by gastric gavage of 200 µL of water for the control group (*n* = 6). Faecal samples were collected on days 1, 6, 8, 10, 14, 16 and 18.

#### 2.6.2. Experiment 2

Throughout the experiment, the drinking water contained 0.5 g·L^−1^ of amoxicillin. Bacterial challenge was performed on day 7. The microencapsulated phage (PEC02) was administered by gastric gavage on days 7, 8 and 9 in eight mice (control group, *n* = 4). Faecal samples were collected on days 1, 6, 8, 10, 12, 16 and 18.

#### 2.6.3. Experiment 3

Mice were exposed to amoxicillin treatment in the drinking water (0.5 g·L^−1^) for the first 8 days. In addition, the drinking water contained pantoprazole (0.1 g·L^−1^) throughout the experiment to facilitate intestinal colonisation and limit phage destruction during gastric transit [17,18]. Bacterial challenge was performed on day 7. From day 14 to 18, mice were treated daily with the phage cocktail by gastric gavage (oral group, *n* = 8) or by gastric and rectal route (oral + rectal group, *n* = 8) or by gastric gavage of 200 µL water for the control group (*n* = 8). Stool samples were taken on days 1, 6, 8, 10, 14 and every 2 days until day 24.

#### 2.6.4. Statistical Analysis

Statistical analyses were performed using the R software (R Foundation for Statistical Computing, Vienna, Austria) version 4.0.0and GraphPad Prism 8.2.1 (GraphPad Software Inc., San Diego, CA, USA). Areas under the curve (AUC) were calculated by the trapezoidal method and compared with a Kruskal-Wallis test. Two-way repeated measures (RM) ANOVA was performed to compare multiple groups, and non-parametric Mann-Whitney tests were performed between groups two-by-two. Negative cultures were fixed at 2 log 10 CFU·g^−1^ of faeces (mean faeces mass was approximatively 50 mg). All tests were defined with an alpha risk determined a priori as significant if 0.05.

## 3. Results

### 3.1. Isolation and Selection of Phages

Out of 20 screened phages, four lytic phages (PEC02, PEC08, PEC16, PEC18) were isolated and selected for their specific activity in vitro against the ESBL E. coli used. When all four were used together in a cocktail they totally inhibited bacterial growth under these in vitro conditions (Figures S2 and S3 in the Data Supplement).

### 3.2. Development of the Murine Model of ESBL E. coli

The experimental designs of the different groups are shown in Figure 1A. The experimental regimen with continuous administration of high-dose amoxicillin (HD) and pantoprazole + amoxicillin HD 8D resulted in higher colonisation (median AUC = 1.09 × 10^10^ and 1.39 × 10^10^ CFU·g^−1^.day, respectively; *p* = 0.70 and *p* < 0.05 compared to the other groups). Continuous administration of amoxicillin HD resulted in lower inter-individual variability between days 3 and 9, with a higher faecal concentration at day 9 (Figure 1B). The addition of pantoprazole resulted in an increase in the initial faecal concentration (day 1 after bacterial challenge) of ESBL *E. coli* compared to amoxicillin HD 8D alone (median = 1.52 × 10^9^ versus 3.25 × 10^7^ CFU·g^−1^; *p* = 0.007). This effect was also still present at day 9 (median = 7.45 × 10^5^ versus 1.81 × 10^3^ CFU·g^−1^; *p* = 0.009), and the median AUC was higher (1.39 × 10^10^ versus 8.83 × 10^7^ CFU·g^−1^.day; *p* = 0.009). However, there was no statistical difference in colonisation between the control and pantoprazole groups (median AUC = 3.06 × 10^4^ versus 3.33 × 10^5^; *p* = 0.06).

### 3.3. In Vivo Evaluation of Phage Therapy

#### 3.3.1. Experiment 1: Oral Cocktail of Phages

**Figure 2:**
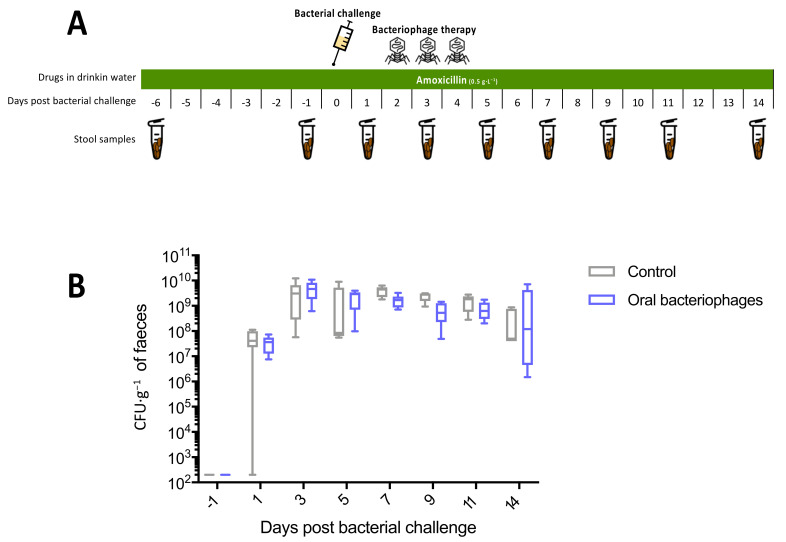
Experiment 1: oral cocktail of phages (**A**), Experimental protocol. (**B**), Faecal concentration of ESBL *E. coli.* Control group (*n* = 6), oral bacteriophages group (*n* = 6).

Figure 2A summarises the experimental procedure. In this experiment, we observed one death in the control group. There was no significant difference in the faecal ESBL *E. coli* concentration between the two groups when total time was taken into account (median AUC = 2.82 × 10^10^ in the control group versus 2.54 × 10^10^ in the treated group, *p* = 0.54; 2-way RM ANOVA, *p* = 0.51). However, in the phage-treated group, we measured a transient decrease in this faecal concentration, starting on days 7 to 11 after bacterial challenge (Figure 2B). This effect was statistically significant on day 9 (median = 5.33 × 10^8^ versus 2.76 × 10^9^ CFU·g^−1^, *p* = 0.02).

#### 3.3.2. Experiment 2: Microencapsulated Phages

**Figure 3:**
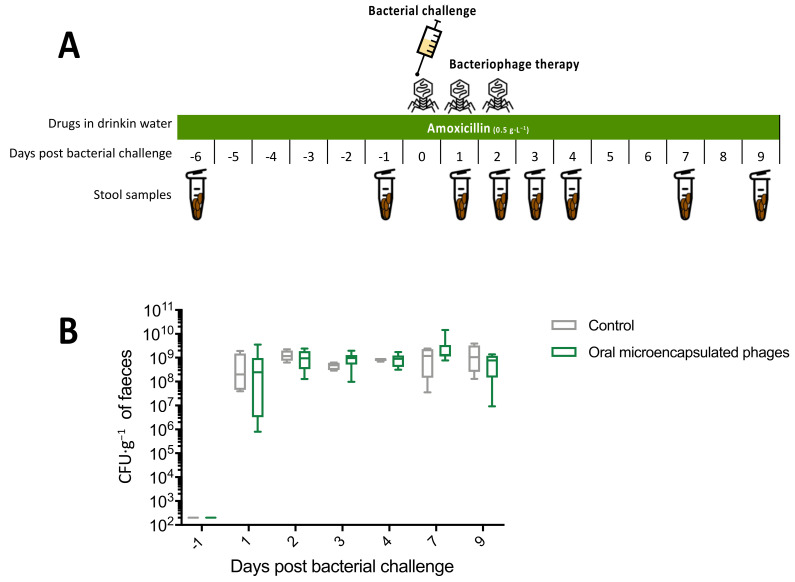
Experiment 2: microencapsulated phages (**A**), Experimental protocol. (**B**), Faecal concentration of ESBL *E. coli.* Control group (*n* = 4), oral microencapsulated phages group (*n* = 8).

The experimental design is illustrated in Figure 3A. There was no significant difference in faecal ESBL *E. coli* concentration between the control and the oral microencapsulated phage groups (median AUC = 8.93 × 10^9^ versus 9.04 × 10^9^ respectively, *p* = 0.81; 2-way RM ANOVA, *p* = 0.44). No transient treatment effect could be observed (Figure 3B).

#### 3.3.3. Experiment 3: Oral and Rectal Cocktail of Phages with Pantoprazole

**Figure 4:**
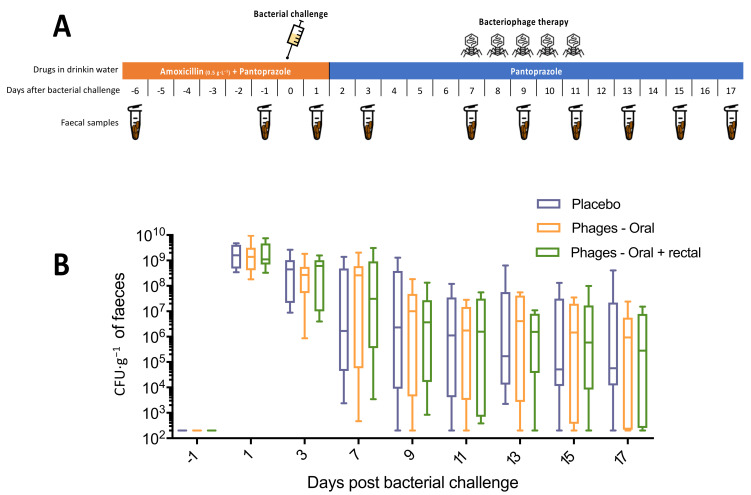
Experiment 3: oral and rectal cocktail of phages with pantoprazole (**A**), Experimental protocol. (**B**), Faecal concentration of ESBL *E. coli.* Placebo group (*n* = 8), oral phages group (*n* = 8), oral and rectal phages group (*n* = 8).

The median and interquartile ranges of ESBL *E. coli* faecal concentrations are presented in Figure 4B. We observed no differences in ESBL *E. coli* concentration between groups, neither for the area under the curve (3.49 × 10^9^, 3.41 × 10^9^ and 3.82 × 10^9^ for the control, oral and oral + rectal groups, respectively; *p*-value > 0.8 for each 2-by-2 group comparison) nor for the 2-way RM ANOVA (*p*-value = 0.96) or the 2-by-2 comparison (Mann Whitney *p*-value > 0.05 for each point).

## 4. Discussion

### 4.1. Summary of Results

In our study, we were not able to show the long-term efficacy of phage therapy in reducing this carriage. We measured a transient reduction in faecal concentration of approximatively 1 log CFU·g^−1^. Nevertheless, we were able to develop several models of intestinal carriage of ESBL *E. coli* with the administration of drugs in drinking water. The advantage of our models was that beta lactam and PPI were diluted in drinking water, which is simpler than some previous models with subcutaneous injections [17,19,20].

### 4.2. Effects of PPI on Digestive Colonisation

We showed that the addition of PPI to antibiotic therapy not only increased the initial faecal ESBL concentration, as shown by Stiefel et al. [17], but also persisted at higher titres away from the bacterial challenge. This facilitating effect of colonisation persistence in our model fits well with observations on humans. Indeed, the meta-analysis of Willems et al. showed an association between the use of acid suppressants and the risk of colonisation by multidrug-resistant microorganisms (MDRO) [21]. The faecal microbiome of PPI users is altered with an over-representation of oral bacteria [22,23]. These alterations in the gut microbiome are the basis for continued MDRO colonisation, but further work is needed to support this hypothesis.

### 4.3. Effectiveness and Limitations of Phages in Reducing Digestive Carriage

In the literature, many phage models aimed at reducing *E. coli* carriage in mice have proven ineffective [15,18,24,25,26] or only transient [27,28,29], regardless of whether phage therapy is administered before or after bacterial challenge, with a single phage or a phage cocktail. The few models that showed efficacy had a treatment protocol that was not considerably different from that of other models without efficacy [15,30,31]. In our model with continuous pressure of antibiotic therapy, we did not observe a lasting efficacy of phage therapy. These results are in agreement with the experiments of Galtier et al. [15], who, in their continuous antibiotic pressure model, found no efficacy. In contrast, a single dose of a cocktail of three phages had good efficacy on the digestive carriage of uropathogenic *E. coli* when antibiotic pressure was no longer present [15]. Proton pump inhibitors also cause changes in the gut microbiota, which may have influenced our results since we did not deal with continuous antibiotic pressure but with continuous pressure by PPI.

The conditions for the optimal efficacy of phage therapy in the gastrointestinal tract are still poorly understood, requiring additional knowledge. Before phages can successfully infect target bacteria in the gut, they must be able to reach and contact them [32]. However, the gut environment can affect bacterial physiology, which may reduce the efficacy of phages compared to in-vitro conditions [33,34,35]. Target bacteria may also reside in the mucosa, which is difficult for phages to access [36]. Another problem is the degradation of phages during intestinal transit and, especially, during gastric passage at low pH [11,18,25]. To counter this phenomenon, we used PPI to increase the gastric pH, in addition to micro-encapsulation of phages. Encapsulation effectively limits the reduction of phage concentration in the gastrointestinal tract after oral administration [37]. Indeed, phages are generally not stable in an environment so acidic as the stomach, some studies use a bicarbonate buffer with oral administration of phages [18,37]. However, the only study on *E. coli* (nalidixic acid-resistant O157:H7) that we found in the literature using encapsulated phages did not show in-vivo efficacy in a bovine model [38]. In contrast, *E. coli* appears to be able to survive for several days in an acidic environment [39]. A limitation of our work regarding the lack of evidence for the efficacy of microencapsulated phages is that we were only able to test it on one phage (PEC02), although this was the most active phage in vitro (Figure in the Supplementary Data). Other animal models have been developed with encapsulated phages to treat *Salmonella* infections, and the results are encouraging [40,41]. However, these infection models are somewhat different from the asymptomatic digestive colonisation models [32].

### 4.4. Limitations of the Study

First, the phages used were sent to us directly by the Clean Cells company and the identification and selection data are only partially available. The precise methods used for in vitro manipulations are not available.

Second, in our study we did not measure the gastric pH or monitor the survival and possible amplification of phages in the digestive tract of mice.

Third, our colonisation model using continuous high dose amoxicillin results in a very high concentration of ESBL *E. coli* which probably reaches a saturation plateau. This model deviates from the colonisation level conditions of human pathology but has the advantage of being stable and homogeneous unlike other models tested where the carriage level was lower over time.

Fourth, a potential transfer of resistance to other bacteria was not specifically evaluated in our study.

Finally, we did not develop a positive model resulting in a significant reduction in ESBL *E. coli* carriage using, for example, antibiotics. Our aim was only to compare phage therapy to placebo.

### 4.5. Perspectives

Finally, phage therapy seems highly attractive for eliminating or reducing the carriage of multidrug-resistant bacteria in the digestive tract because of its specificity, its low impact on the digestive microbiome and its safety. However, the in-vivo results are, so far, rather dissatisfying, except for some infectious models such as cholera [32]. Moreover, the only human study of intestinal phage therapy showed disappointing results; this clinical trial that attempted to treat *E. coli* diarrhoea in children failed to achieve replication of intestinal phages and improved outcomes [42]. There are still many obstacles to be elucidated in order to be able to implement this therapy in routine practice.

## 5. Conclusions

Oral treatment with amoxicillin was able to promote digestive carriage in mice, which was also the case for the addition of PPI. However, our study confirms the difficulty of achieving efficacy with intestinal phage therapy to reduce MDR bacterial carriage in vivo. Only a small transient effect could be observed in one experiment, while the other experimental conditions showed no effectiveness. Many obstacles need to be further investigated and better understood before this approach can be used in routine practice in this indication.

## Data Availability

The datasets generated during and/or analysed during this study are available from the corresponding author upon reasonable request.

## Supplementary Materials

The following are available online at https://www.mdpi.com/article/10.3390/microorganisms9122580/s1. Figure S1. ESBL E. coli on chromatic ESBL agar plates (pink to burgundy coloration). Figure S2. Susceptibility of ESBL E. coli to phages tested. Figure S3. Monitoring of ESBL E. coli growth by OD 600nm reading on a 96-well plate automat.
