# Supplementary material for: Impact of Phage Therapy on Multidrug-Resistant Escherichia coli Intestinal Carriage in a Murine Model
Source: Microorganisms. 2021 Dec 13;9(12):2580. doi: 10.3390/microorganisms9122580 (PMC8708983; doi:10.3390/microorganisms9122580)
Supplement: Supplementary file 1 [file microorganisms-09-02580-s001.zip › microorganisms-1479298-supplementary.pdf]

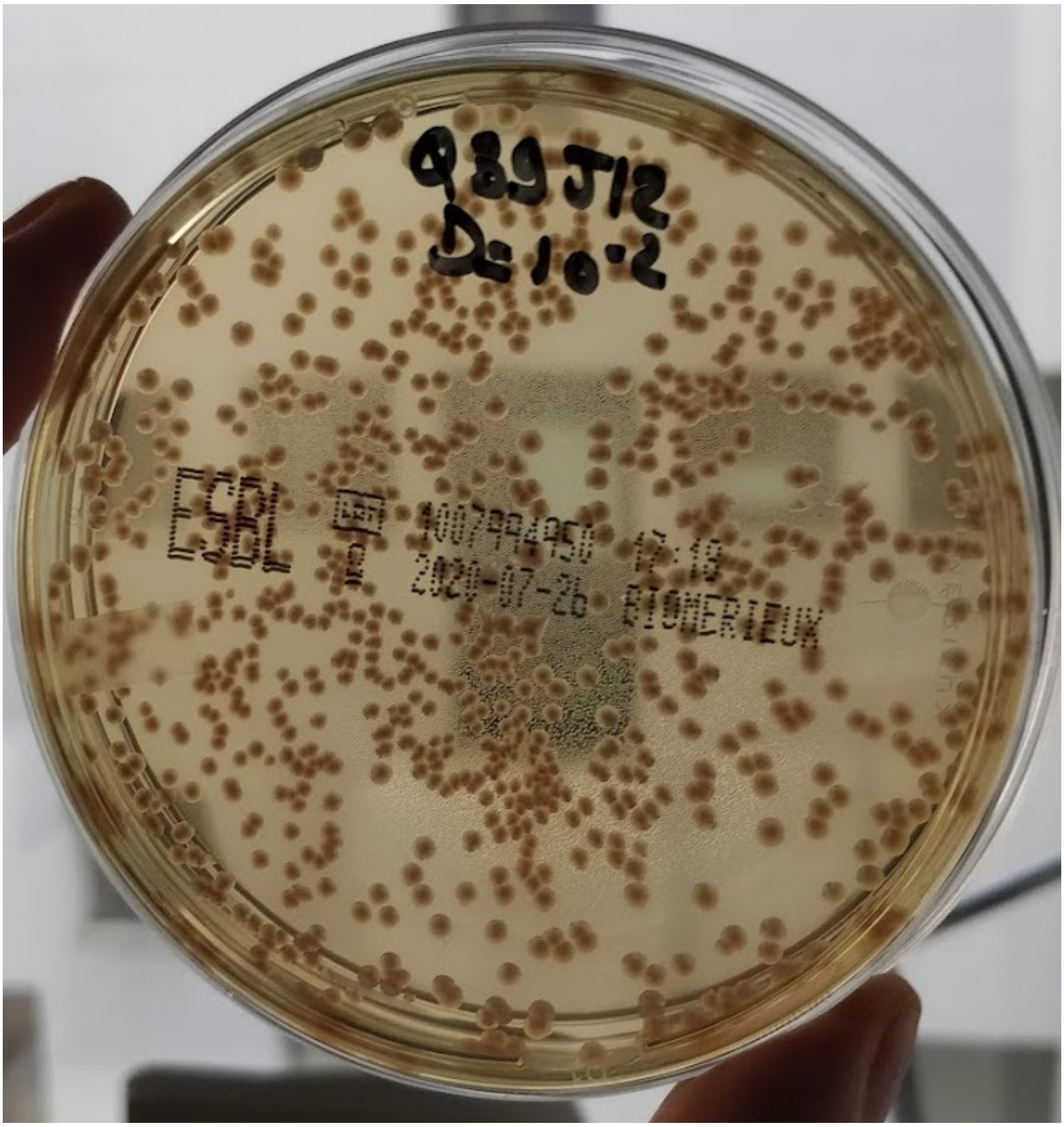

Figure S1. ESBL *E. coli* on chromatic ESBL agar plates (pink to burgundy coloration)

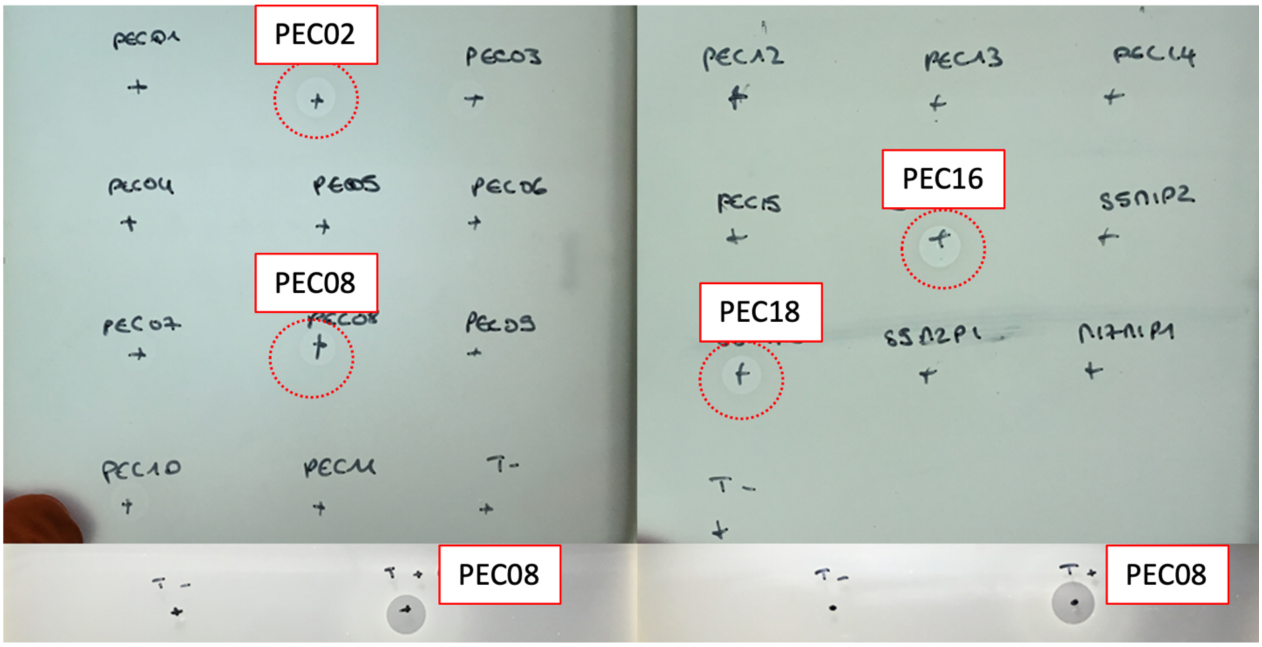

Figure S2. Susceptibility of ESBL *E. coli* to phages tested

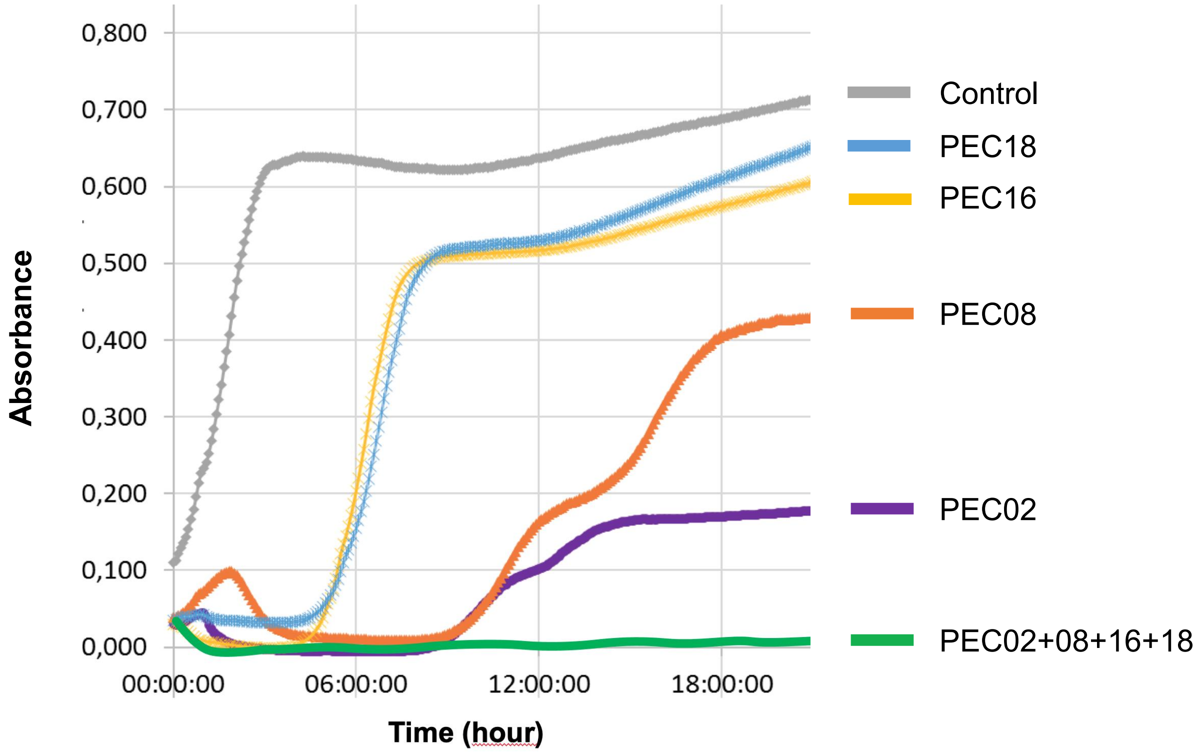

Figure S3. Monitoring of ESBL E. coli growth by OD 600nm reading on a 96-well plate automat.
